# Long term *ex vivo* culturing of *Drosophila* brain as a method to live image pupal brains: insights into the cellular mechanisms of neuronal remodeling

**DOI:** 10.3389/fncel.2015.00327

**Published:** 2015-08-24

**Authors:** Dana Rabinovich, Oded Mayseless, Oren Schuldiner

**Affiliations:** Department of Molecular Cell Biology, Weizmann Institute of SciencesRehovot, Israel

**Keywords:** *Drosophila*, neuronal remodeling, mushroom body, brain culturing, metamorphosis, axon pruning, live-imaging

## Abstract

Holometabolous insects, including *Drosophila melanogaster*, undergo complete metamorphosis that includes a pupal stage. During metamorphosis, the *Drosophila* nervous system undergoes massive remodeling and growth, that include cell death and large-scale axon and synapse elimination as well as neurogenesis, developmental axon regrowth, and formation of new connections. Neuronal remodeling is an essential step in the development of vertebrate and invertebrate nervous systems. Research on the stereotypic remodeling of *Drosophila* mushroom body (MB) γ neurons has contributed to our knowledge of the molecular mechanisms of remodeling but our knowledge of the cellular mechanisms remain poorly understood. A major hurdle in understanding various dynamic processes that occur during metamorphosis is the lack of time-lapse resolution. The pupal case and opaque fat bodies that enwrap the central nervous system (CNS) make live-imaging of the central brain *in-vivo* impossible. We have established an *ex vivo* long-term brain culture system that supports the development and neuronal remodeling of pupal brains. By optimizing culture conditions and dissection protocols, we have observed development in culture at kinetics similar to what occurs *in vivo*. Using this new method, we have obtained the first time-lapse sequence of MB γ neurons undergoing remodeling in up to a single cell resolution. We found that axon pruning is initiated by blebbing, followed by one-two nicks that seem to initiate a more widely spread axon fragmentation. As such, we have set up some of the tools and methodologies needed for further exploration of the cellular mechanisms of neuronal remodeling, not limited to the MB. The long-term *ex vivo* brain culture system that we report here could be used to study dynamic aspects of neurodevelopment of any *Drosophila* neuron.

## Introduction

Developmental neuronal remodeling is an essential step in the proper development of both vertebrate and invertebrate nervous systems. Development of precise neuronal circuits often involves the formation of exuberant connections followed by degenerative events, such as neurite pruning, that may be subsequently followed by regrowth and the formation of new, adult specific, connections (Luo and O'Leary, 2005; Schuldiner and Yaron, 2015). Axon fragmentation during developmental axon pruning shares some mechanistic similarities with axon fragmentation and elimination in neurodegenerative diseases or adjustment of neuronal connections in response to injury (Hoopfer et al., 2006; Saxena and Caroni, 2007). Therefore, understanding the molecular and cellular mechanisms governing neuronal remodeling should provide us with a broader understanding of the mechanisms regulating axon fragmentation and elimination during development and disease. Despite recent advancements, the cellular mechanisms that regulate these processes are not fully understood.

The stereotypic developmental remodeling of *Drosophila* mushroom body (MB) γ neurons is an attractive model system to uncover the molecular and cellular mechanisms underlying neuronal remodeling (Lee et al., 1999). The *Drosophila* MB is derived from four identical neuroblasts that sequentially give rise to three major classes of neurons, among them only one class, the γ neurons, undergo remodeling (see **Figure 2A**). During the larval stages, MB γ neurons send bifurcated axons projecting both medially and dorsally. At the onset of metamorphosis, γ neurons prune their dendrites completely and their axons up to a specific location that coincides with the original branch-point. Later during development, γ neurons regrow their axons to an adult-specific medial lobe (Yaniv et al., 2012). Despite detailed morphological characterization (Lee et al., 1999), we still have a poor understanding of the dynamic cellular mechanisms that govern neuronal remodeling. For example, while Watts et al. (2003) showed that axon pruning of MB γ neurons occurs by localized fragmentation, the precise sequence of events is still unknown.

The main reason for our limited knowledge is the lack of time-lapse imaging of MB neuronal remodeling. In contrast to class IV dendrite arborization sensory neurons (C4da, also known as da neurons), where the superficial soma and dendritic projections have enabled time-lapse imaging during pupal development (Williams and Truman, 2004) MB and other CNS neurons reside deep within the pupa. Imaging the pupal brain *in vivo* is not possible because of the pupal cuticle and even more importantly, an opaque layer of fat bodies that enwrap the brain at these stages and prevents detection of fluorescent signals from the central nervous system in a living animal.

Recent advancements in the field of culturing larval (Siller et al., 2005) pupal (Gibbs and Truman, 1998; Prithviraj et al., 2012; Zschätzsch et al., 2014) and adult (Ayaz et al., 2008) brains, encouraged us to test whether we can image neuronal remodeling processes in whole brain explants. So far, *ex vivo* culture methods that allow high-resolution analysis of the dynamics of developmental processes have described only short-term (up to 1 h) live imaging of the central brain (Siller et al., 2005; Zschätzsch et al., 2014; Medioni et al., 2015). Because remodeling of MB neurons occurs on the time scale of hours, it was necessary to modify and optimize existing methods.

We present here a method for long term *ex vivo* pupal brain culturing that permits normal brain development, as well as time-lapse imaging of neuronal remodeling. This method should be useful to investigate various dynamic aspects of remodeling as well as allow pharmacological manipulations of brains in culture. Additionally, the method is not limited to MB neurons and should thus allow time-lapse imaging of other neuronal processes that take place in the brain during the pupal stage. We used this technique to analyze the cellular mechanism by which γ axons undergo fragmentation, and found that axon fragmentation is initiated in parallel in one to two different locations in each axon and that different axons within the same bundle undergo fragmentation in different kinetics.

## Methods

### Drosophila genotypes

**Figures 2B–E, 4C,D**: 201Y-Gal4, UAS-mCD8::GFP
**Figures 3H,I, 4E**: 201Y-Gal4, UAS-mCD8::GFP/+; UAS-EcR-DN/+
**Figure 5A**: 201Y-Gal4, UAS-mCD8::GFP/+; UAS-mCD8::RFP/+
**Figure 5B**: 201Y-Gal4, UAS-mCD8::GFP/+; UAS-mCD4::Tomato/+
**Figure 6**: hsFLP, UAS-mCD8::GFP/+;GMR82G02-Gal4, UAS-mCD8::RFP, FRT40A, Gal-80/FRT40A

### Generation of MARCM clones

Mushroom body (MB) MARCM neuroblast clones were generated at newly hatched larva (NHL) and examined later, as described previously (Lee et al., 1999).

### Antibody staining conditions

Rat monoclonal anti-mouse CD8 α subunit, 1:100 (Invitrogen); mouse monoclonal anti-FasII (1D4), 1:25 (Developmental Studies Hybridoma Bank). Alexa 488 or Alexa 647 conjugated secondary antibodies were used at 1:300 (Invitrogen). Fixed brains were mounted on Slowfade (Invitrogen) and imaged using a Zeiss LSM710 confocal microscope.

### *Ex Vivo* brain culturing system

Pupae were collected at puparium onset (white pupae) and aged until the required developmental stage at 25°C. All equipment (forceps, wells etc.) was disinfected using 70% ethanol prior to dissections. The brains were quickly (< 2 min/brain) and carefully dissected using conventional method (Wu and Luo, 2006) in a filtered and sterile basic culture medium: Schneider's insect media (Biological Industries) supplemented with 10% heat inactivated FBS, 1:100 100XAntibiotic-Antimyotic (Gibco), 0.5 mM Ascorbic acid (Sigma) and 20-hydroxyecdysone (Sigma-Aldrich) at the appropriate concentration as described in the results section. Media supplements were prepared as concentrated stocks and frozen in small aliquots. Thawed aliquots were not refrozen. All the brains for a single experiment were dissected simultaneously in the basic media, and were randomly divided to wells containing modified media according to the conditions and controls tested. In experiments that required a large amount of brains, several people dissected simultaneously in order to minimize timing differences. Damaged brains were discarded. Cultured brains were kept in a standard fly incubator at 25°C for the number of indicated hours, covered with a semi-permeable membrane to prevent contamination (YSI incorporated, LN# 13A 100487) until fixation and processing for immunohistochemistry according to standard protocols.

### Time-lapse imaging and processing of **γ** axons remodeling

Confocal imaging was performed using Zeiss LSM 710 Confocal microscope (Zeiss), with a X40 oil objective. Two-photon imaging was performed using a Zeiss LSM 7 MP upright microscope (Zeiss) with a W-Plan Apochromat X20 objective, NA 1.0.

For time-lapse imaging using confocal microscopy, brains were mounted in glass bottomed dishes (Mattek). In some of the experiments we restricted the lateral movement of the brains by manually making brain sized (900 μm) holes in Polydimethylsiloxane (PDMS) that was then attached to the imaging dishes. To make a PDMS sheet, PDMS was mixed at 10:1 weight ratio; 10 ml were poured on 7.5 cm diameter silicon template and spun at 1500 rpm for 30 min. PDMS was baked for 30 min at 80°C until PDMS is fully cured. After curing, the PDMS layer was cut to stripes, peeled, perforate using 900 μm needle and gently placed on a glass bottomed dish (modified from Zaretsky et al., 2012).

For two-photon microscopy, mounting was done on a circular custom imaging chamber that contained an additional circular chamber within (inner diameter-2 cm, outer diameter-5 cm Nicenboim et al., 2015). The inner circle of the imaging chamber was filled with 2% UltraPure LMP Agarose (Invitrogen 16520-050) before dissections to allow the agarose to solidify. The chamber was filled with culturing media. Immediately following dissections a small hole (brain sized) was made in the agar using forceps and the brain was gently inserted into it (see detailed Supplementary Protocol). Imaging was performed immediately using a two-photon microscope and GaAsP detectors. Excitation of GFP was carried out at 920 nm, co-imaging of GFP and a red fluorophore was performed using an excitation wavelength of 910 nm. The laser intensity was kept to as low as possible in order to avoid bleaching. *z*-stacks were acquired at 1 μm increments, every 5–20 min. For multi specimen imaging the Zen MTS macro was used. Images were processed using Fiji (NIH) for max projection and time-lapse. Minor movement of the brain during the imaging was corrected using Fiji “Stack Reg” plugin. For single-cell imaging processing selected data sets were deconvoluted with Autoquant X3 (Media Cybernetics), cropped, and registered using Fiji plugin “correct 3D drift” and later analyzed in Imaris (Bitplane) for 3D rendering and iso surface creation.

### Quantification and statistical analyses

For quantification of MB axon pruning *ex vivo* we measured the sum intensity of an ROI at the branch point and at the dorsal tip of confocal lsm files using Fiji (see **Figure 2F**). To calculate the ratio we then divided the sum of intensity of the dorsal tip by that of the branch point, and by that calculated the pruning index (i.e., ratio of about 1 represent completely unpruned lobe, a ratio close to 0 represent complete pruning, and anything in between represent partial pruning). Statistical analysis was performed by a two-tailed independent sample *T*-test in **Figure 3J** or by ANOVA including all groups with a two-tailed Dunnett *post-hoc* in **Figures 2E, 3K**.

## Results

### *Ex vivo* brain culture recapitulates developmental morphological changes

**Figure 1:**
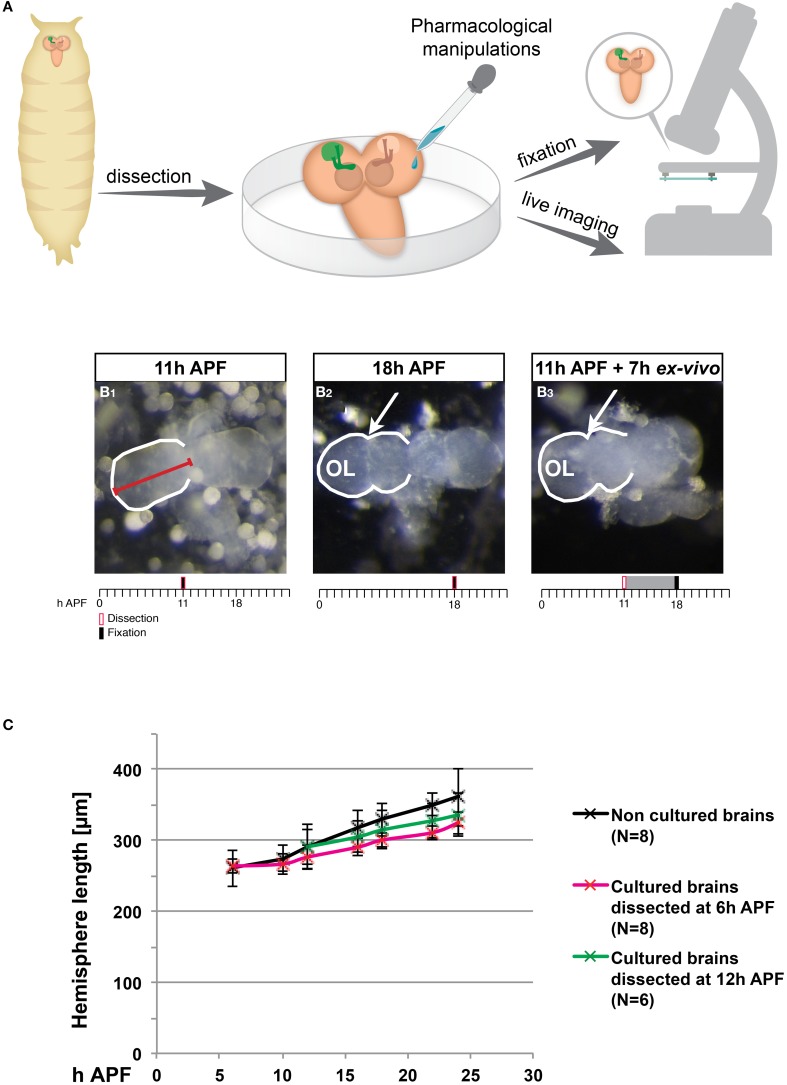
***Ex vivo* cultured brains undergo morphological changes that are similar to normal development. (A)** Scheme of the *ex vivo* culturing procedure. Brains were dissected from pupae at various developmental times and cultured, at which time they could have been manipulated pharmacologically, time-lapse imaged or fixed and stained with specific antibodies. **(B_1_–B_3_)** Stereoscope images of brains dissected and imaged at 11 h after puparium formation (APF) **(B_1_)** or 18 h APF **(B_2_)** or brains dissected at 11 h APF and imaged after 7 h in culture **(B_3_)**. Red line marks hemisphere length. The ridge-like shape that is part of the developing optic lobe is marked with arrows. Timeline provides information regarding the timing of dissection (red box), fixation (black bar) and culturing (gray interval). **(C)** Quantification of the change in hemisphere length in brains fixed immediately following dissections (black series), and in brains dissected at 6 h APF (red series) or 12 h APF (green series) followed by culturing. An increase in hemisphere length is observed both *in vivo* and in cultured brains for the first 18 h of culturing.

To determine whether cultured pupal brains continue to undergo normal development, we dissected brains at an early pupal stage and cultured them in an insect growth media for extended periods of time (1–4 days; Figure 1A). We observed that, on a gross level, many of these cultured brains exhibited morphological changes that resembled normal development. During early pupa, the brain undergoes distinct morphological changes, due to massive tissue remodeling and growth (Figure 1, compare Figures 1B_1_,B_2_). Up until about 11 h after puparium formation (APF) the brain retains spherical hemispheres similar to the larval brain (Figure 1B_1_), and slowly increases in size. From about 12 h APF until 18 h APF the brain loses its characteristic spherical shape and begins to elongate, and in parallel the adult optic lobes begin to develop, resulting in a ridge-like hemisphere shape (arrow in Figure 1B_2_). Remarkably, this loss of sphericity as well as optic lobe development was also observed in brains that were dissected at 11 h APF and cultured for 7 h *ex vivo* (Figure 1B_3_). In order to quantify and compare the development of brains *in vivo* compared to *ex vivo*, we measured hemisphere length (see red line in Figure 1B_1_). During normal development the hemisphere length increases by about 40% between 6 and 24 h APF (Figure 1C also see Supplemental Movie 1). We found that cultured brains dissected at 6 h or at 12 h APF displayed an increase in hemisphere length that was similar to what occurred during normal development although they exhibited a slight delay in growth following several hours in culture. These observations suggest that pupal brains continue to develop in culture.

### Pruning of mushroom body (MB) **γ** axons proceeds normally in *Ex Vivo* cultured brains

**Figure 2:**
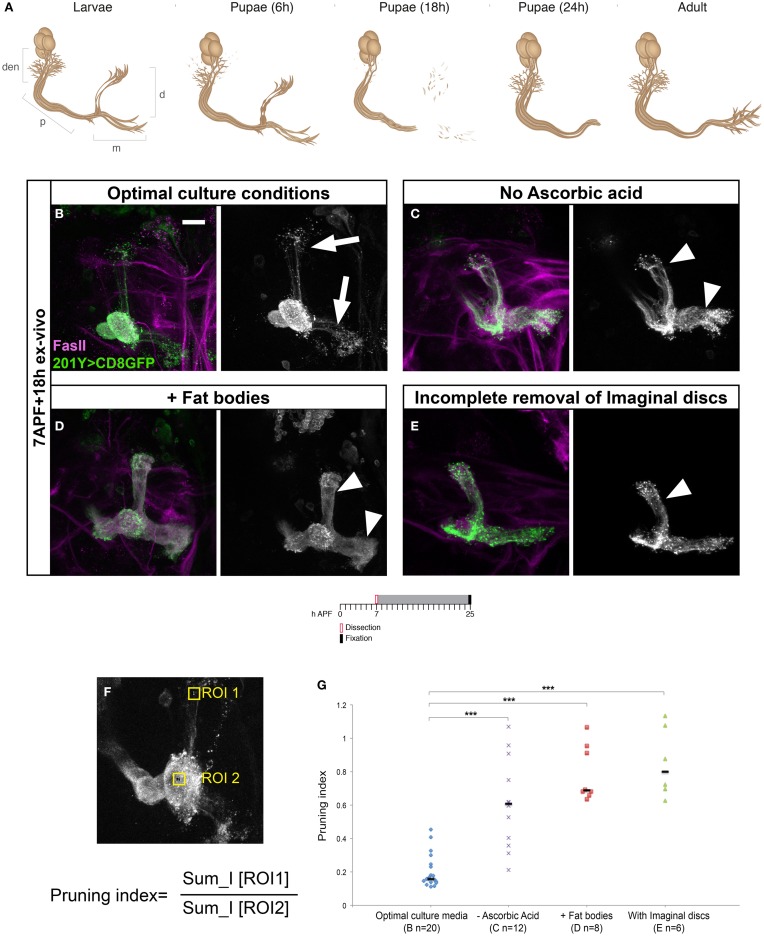
**Specific culturing conditions are critical for maximizing *ex vivo* development of dissected brains. (A)** Schematic representation of MB γ neuron remodeling. Den, dendrites; p, peduncular axon; m, medial; d, dorsal, h, after puparium formation (APF). **(B–D)** Confocal Z-projections of MBs labeled by mCD8-GFP driven by Gal4-201Y (green) and stained for FasII (magenta) of brains dissected at 7 h APF and cultured for ~18 h with optimal conditions **(B)** or without ascorbic acid **(C)**, in the presence fat bodies **(D)**, and with imaginal discs **(E)**. **(F)** Confocal Z-Projection of MB labeled by mCD8-GFP driven by Gal4-201Y, with two regions of interest (ROI) marking the axonal tip (ROI 1) and the branching point (ROI 2). Pruning Index was calculated as the ratio of the sum intensities of ROI1/ROI2. **(G)** Quantification of axon pruning *ex vivo* of the conditions shown in **(B–D)** using a two-tailed ANOVA followed by Dunnett's *post-hoc* test, ^***^p < 0.001. Vertical black lines represent the median. The scale bars represent 20 μm. Arrowheads demarcate unpruned axons, magenta arrows mark pruned lobes. While the addition of ascorbic acid significantly improved pruning *ex vivo* (**B**, compare to **C**), the presence of fat bodies **(D)** or imaginal discs **(E)** resulted in impaired pruning.

We then wanted to see if developmental axon pruning occurs in culture. At the larval stages MB γ neurons send a bifurcated axon that during metamorphosis undergoes pruning by localized fragmentation up to the branch point and later regrows to adult specific areas (Figure 2A). Morphological aspects of pruning, including microtubule disassembly and membrane blebbing, become visible at around 6 h APF and axons become completely fragmented by 18 h APF (Watts et al., 2003). Using MB development as a read-out, we first decided to optimize media conditions to maximize *ex vivo* development of the dissected brains. We therefore tested the effect of several media additives for their influence on the ability of γ axons to prune *ex vivo*, among them insulin, glutamic acid, and ascorbic acid that were suggested to be required in other organ culturing systems (Siller and Doe, 2008). While the presence of insulin or glutamic acid had no effect on γ axon pruning in culture (data not shown) the supplement of ascorbic acid to the culturing media significantly improved proper pruning of MB neurons in culture (compare Figures 2B,C, quantified in Figure 2G, see Figure 2F and materials and methods for quantification method). In addition to optimizing the concentration of different additives to the culture media, we tested whether the addition or exclusion of pupal fat bodies affected development as previously suggested (Sousa-Nunes et al., 2011). To our surprise, and in contrast to previous indications, we found that culturing brains with fat bodies derived from the same dissected pupaes, significantly inhibited development of the brains rather than promoting development (Figure 2D, quantified in Figure 2G). We also found that removing the imaginal discs completely during dissection, dramatically improved brain development (compare Figures 2B–E, quantified in Figure 2G). Since damage to the imaginal discs was found to retard metamorphosis (Stieper et al., 2008), a reasonable explanation for the negative effect of leaving the imaginal discs could be that the imaginal discs are injured during the dissection and thus may inhibit *ex vivo* development, unless they are completely removed.

**Figure 3:**
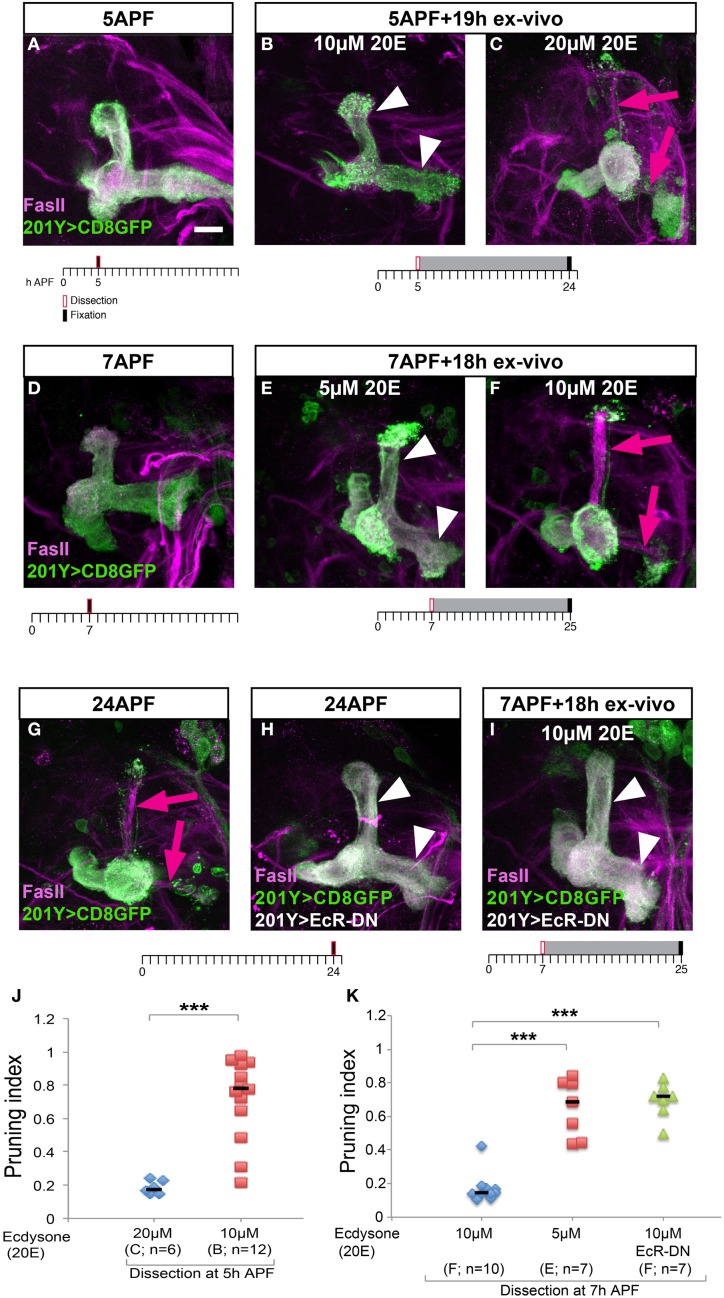
**Axon pruning in culture requires differential ecdysone levels, which depends on the age of the pupa. (A–I)** Confocal Z-projections of MBs stained for FasII (magenta) and labeled by 201Y-Gal4 driving the expression of membrane bound GFP (mCD8-GFP) in γ neurons. In **(H,I)**, expression of a dominant negative version of the Ecdysone receptor (EcR-DN) was also driven by 201Y-Gal4. Brains were dissected and fixed at 5 h APF **(A)**, 7 h APF **(D)**, 24 h APF **(G,H)**, or cultured for 18 or 19 h after dissection at 5 h APF **(B,C)** or at 7h APF **(E,F,I)**. The scale bars represent 20 μm. Arrowheads demarcate unpruned axons, arrows mark newly grown α/β. **(J)** Quantification of axon pruning *ex vivo* of the conditions shown in **(B,C)** using a two-tailed independent sample *T*-test, ^***^p < 0.001. **(K)** Quantification of axon pruning *ex vivo* of the conditions shown in **(E,F,I)** using a two-tailed ANOVA followed by Dunnett's *post-hoc* test, ^***^p < 0.001. Vertical black lines represent the median. While brains dissected at 7 h APF require 10 μM 20-hydroxyecdysone (20E) for proper pruning *ex vivo* **(F)**, brains dissected at 5 h APF require 20 μM 20E to sustain pruning **(C)**. MBs expressing EcR-DN fail to prune γ axons in culture **(I)**, as they do *in vivo* **(H)**.

The major effect on pruning efficiency in cultured brains, however, resulted by manipulation of the levels of the major *Drosophila* steroid hormone—ecdysone (20-hydroxyecdysone; 20E). First, it is important to mention that despite numerous attempts of culturing the brains in varying levels of ecdysone and insulin, we were unable to identify culture conditions that supported brain development and pruning in brains that were dissected at the onset of metamorphosis (0 h APF) suggesting that the culture system cannot completely recapitulate all the developmental signals in early pupa. However, and in line with the known ecdysone peak at the onset of puparium formation (Richards, 1981), we found that brains dissected during early metamorphosis required higher 20E concentration than those dissected at later time points. More specifically, in brains dissected at 5 h APF and cultured for 19 h, addition of 20 μM 20E was sufficient to promote normal development but 10 μM 20E was not (Figures 3A–C, quantified in Figure 3J). In contrast, in brains dissected at 7 h APF and cultured for 18 h, 10 μM 20E was sufficient to induce pruning but lowering 20E concentrations even further to 5 μM was not (Figures 3D–F, quantified in Figure 3K). Interestingly, the later born α/β MB neurons (strongly labeled by FasII antibody staining) also extended their axons during the culture process (magenta arrows in Figures 3C,F, compare to the equivalent uncultured brain in Figure 3G) in the appropriate developmental time window, providing another indication to the proper *ex vivo* development of the MB as a whole. Nonetheless, to prove that the γ axons indeed prune, and not spontaneously degenerate due to culturing conditions, we exploited the fact that the Ecdysone receptor B1 (EcR-B1) is cell autonomously required in these neurons for remodeling. Therefore, we expressed a dominant negative form of EcR (EcR-DN) in the MBs of the cultured brains and tested whether they pruned in culture. Indeed, brains expressing EcR-DN did not undergo pruning in culture (Figure 3I, quantified in Figure 3K) or *in vivo* (Figure 3H) suggesting that the axon fragmentation we observed during culturing was not due to culture-induced degeneration and therefore likely resembles pruning during normal development.

### Culturing pupal brains *Ex Vivo* allows for time-lapse imaging of axon pruning

**Figure 4:**
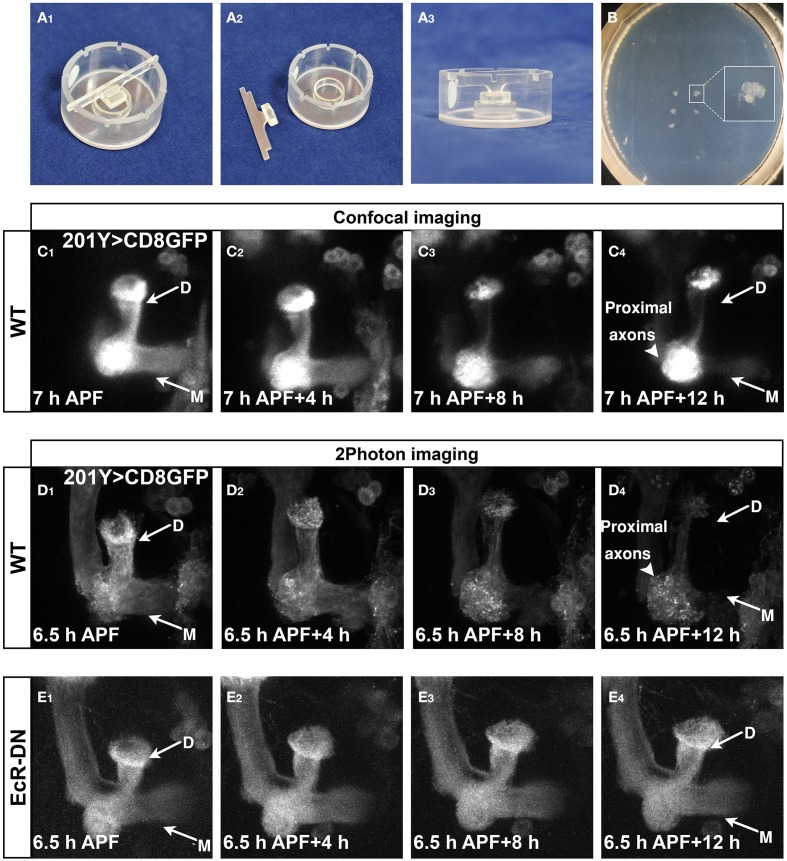
**Developmental processes, such as pruning, can be time-lapse imaged in culture. (A,B)** Mounting setup **(A)** and mounted brains **(B)**, see more in the methods section and Supplemental Protocol. **(C–E)** Confocal **(C)** or two-photon **(D,E)** Z-stacks of images extracted from time-lapse movies of MBs labeled by mCD8-GFP driven by Gal4-201Y **(C,D)** or additionally expressing EcR-DN **(E)** in brains dissected at 6.5–7 h APF and cultured for 12 h (first time point—**C–E_1_**, last time point—**C–E_4_**). Axons projecting to the dorsal and medial γ lobes were eliminated during the culturing of WT MBs (arrows in **C_4_,D_4_**), while the cell bodies and the proximal part of the axons remain intact (arrowheads in **C_4_,D_4_**). In brains expressing EcR-DN axon elimination was inhibited and the axons remain intact throughout the imaging (arrows in **E_4_**). D, Dorsal lobe; M, medial lobe. See also Supplemental Movies 2–4.

We next wanted to utilize this *ex vivo* culture system for time-lapse imaging of MB γ axon pruning. To this end, we cultured brains expressing membrane bound GFP (mCD8-GFP) in MB neurons (driven by the γ neurons specific driver 201Y-Gal4) and time-lapse imaged them using a confocal or two-photon microscopes. Although seemingly a simple extension of the brain culturing, this was technically challenging, as we had to find a way to prevent the brains from drifting out of the frame during imaging. We first tried using biological glues such as polylysine and laminin, as previously used by other (Brown et al., 2006; Williamson and Hiesinger, 2010; Zschätzsch et al., 2014; Medioni et al., 2015), but we found out that while they do efficiently fix brains onto the plate, the development of these brains was dramatically retarded (data not shown). After trying several simple and more complex options, we selected different solutions for an inverted confocal microscope and a two-photon microscope. When we imaged using inverted confocal microscopy, we placed single brains in a 96-well glass bottomed dish. In some experiments, we restricted the lateral movement of the brains by using a Polydimethylsiloxane (PDMS) mold attached to the imaging dishes (see Methods). While this solution was somewhat helpful in imaging brains in the inverted confocal microscope, it was not suitable for imaging in an upright microscope because the movements of the dipping lens created ripples that agitated the brains. After trying several options, we found that a relatively “low tech” solution of sinking brains in pre-made indentations in low melting agar worked best for imaging in the two-photon microscope (Figures 4A,B, modified from Nicenboim et al. (2015), for more details see Supplementary Protocol). By using these techniques, the brains remain relatively stationary during imaging and continue to develop normally. While imaging using an inverted confocal microscope (LSM710) resulted in fast bleaching and low resolution (Figure 4C, Supplemental Movie 2), imaging using a two photon microscope (LSM 7 MP) resulted in better resolution and lower bleaching throughout the time lapse session (Figure 4D, Supplemental Movie 3). Due to its superior imaging capabilities, we used the two-photon platform in our subsequent experiments. However, we cannot rule out that using a confocal microscope with better detectors and/or more stable fluorophores might also be sufficient. At the beginning of the imaging session (6.5–7 h APF) both dorsal and medial lobes of the γ lobe were clearly observed (arrows in Figures 4C_1_,D_1_). After 12 h in culture most of the axons were eliminated (arrows in Figures 4C_4_,D_4_) up to the branch point (arrowheads in Figures 4C_4_,D_4_), while the cell bodies (out of frame) and the proximal parts of the axons remained intact. Consistent with the culturing data (Figures 3G–K), live imaging of brains expressing EcR-DN within MB neurons resulted in static time-lapse imaging and failure of the γ axons to undergo pruning (Figure 4E and Supplemental Movie 4).

**Figure 5:**
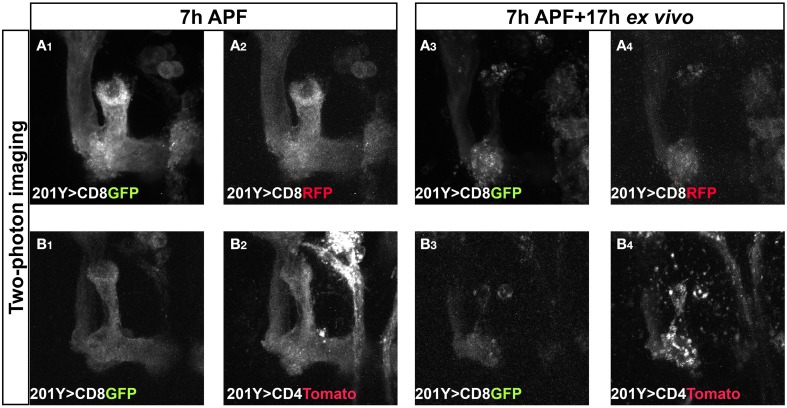
**Two color, two-photon imaging reveals different usefulness of various red fluorescent proteins**. Two-photon Z-stacks of images extracted from a time-lapse movie of MBs labeled by mCD8-GFP and either mCD8-RFP **(A)** or mCD4-Tomato **(B)** in brains dissected at 7 h APF and cultured for 17 h. Time-lapse imaging of mCD4-Tomato **(B)** resulted in puncta during the imaging (compare **B_4_** to **B_2_,B_3_**). mCD8-RFP provides good anatomical representation throughout the imaging similar to mCD8-GFP (compare **A_4_** to **A_2_,A_3_**). See also Supplemental Movies 5, 6.

We then wanted to explore the possibility of imaging two fluorescent proteins concurrently. While this is rather simple in single photon microscopy, imaging of multi-channel images is not always straightforward in two-photon microscopy, where the dynamic range of excitation is more limited. This is especially true in live samples, since there is no time to switch between two excitation wavelengths and limited overlap in the excitation wavelengths of GFP and RFPs. However, some red fluorescent proteins exhibit secondary excitation peaks that coincides with GFP (Drobizhev et al., 2011). We therefore tested several red fluorophores using both confocal (data not shown) and two-photon microscopes and found that some of them produced puncta during imaging for unknown reasons (CD4-Tomato, Myr-RFP, Figure 5B, and data not shown, and Supplemental Movie 5), thus limiting their usefulness. The only red fluorescent protein, which we tested, that provided good anatomical representation throughout live imaging is mCD8-RFP (Figure 5A and Supplemental Movie 6). Whether these differences are an inherent property of the fluorescent proteins or the membrane attachment tether still needs further exploration. While we cannot rule the possibility that these red puncta are a result of incomplete degradation of the fluorescent protein, as RFPs were previously shown to be stable in a wider pH range than GFPs (Han et al., 2014), this is unlikely because the GFP seems intact and the mCD8-RFP also yielded uniform staining. Therefore, for future imaging of two channels at the same time we recommend using mCD8-RFP or testing the reliability of various red fluorescent proteins in order to avoid artifacts.

### Axon fragmentation of MB axons is initiated with a proximal axonal break

**Figure 6:**
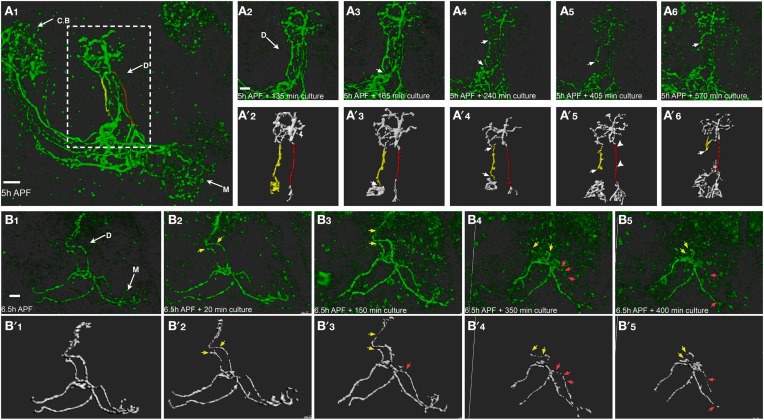
**Time lapse imaging in a single cell resolution allows a careful examination of the cellular mechanisms of remodeling**. Two-photon Z-stacks of selected images extracted from a time-lapse movie of multi-cell clone **(A)** or two-cell clone **(B)** labeled by mCD8-GFP driven by Gal4-GMR82G02. **(A)** Different axons within the same bundle undergo fragmentation in different kinetics. While the lateral axon (marked in yellow) is already fragmented (arrows in **A_3_**), the more medial axon (marked in red) only begins to bleb (arrowheads in **A_5_**). Lower panels represent ISO-surface rendering of the fluorescent data (for detailed image processing see Methods and Supplemental Movie 8). **(B)** Axons projecting to the dorsal γ lobe were eliminated at an early time point (yellow arrows) compared to the medially projecting axons (red arrows). Arrows mark nicks formation and fragmentation progression, arrowheads represent blebbing. CB, cell bodies; D, Dorsal lobe; M, medial lobe. The scale bars are 40 μm.

While imaging of brains that express mCD8-GFP in the entire MB resulted in the first time-lapse movies of remodeling, they do not provide single cell resolution. We therefore used the MARCM technique to create multiple single-cell clones using a specific MB γ neuron driver (GMR82G02-Gal4, obtained from the FlyLight collection). Using this strategy we imaged pruning of single MB γ axons using two-photon microscopy (Figures 6A,B shows selected frames obtained in Supplemental Movies 7, 8, respectively). Live-imaging of single axon resulted in weaker signal and higher background noise compared with neuroblast clones, therefore requiring more detailed image analysis (see Methods and Supplemental Movie 9). While at 5 and 6 h APF the axons appear continuous (Figures 6A_1_,B_1_), after 2–4 h of imaging they become blebbed (Figure 6A, which is focused on the dorsal lobe, compare red and yellow tracked axons in Figures 6A_2_,A_4_). This is similar to what is observed *in vivo*, indicating the onset of degeneration. After the “blebbing” phase, the axon becomes fragmented at 1–2 locations in parallel, in what seems to be random locations (arrows in Figures 6A_3_,A_4_,B_3_). Following axon severing, the remaining axon becomes swelled and then degenerates in what seems to be a rapid and uniform process (arrow in Figures 6A_5_,A_6_). The whole process occurs within 6 h of imaging. Interestingly, different axons within the same bundle undergo fragmentation in different kinetics, as two distinct axonal processes within one multi single-cell clone fragmented at different time scale (Figure 6A). While one axon is already fragmented (arrow in Figure 6A_3_), the more medial axon (marked in red) only begins to bleb (arrowheads in Figure 6A_3_), thus suggesting a cell-autonomous process. However, we cannot rule out the possibility that neighboring glia play a role in these severing events. The fragmentation dynamics of the dorsal lobe compared to the medial lobe is also asynchronous. Figure 6B shows an example in which the dorsal lobe degenerates at earlier time points than the medial lobe. In this example, while the dorsal projections are almost completely pruned up to the branch point (yellow arrows in Figure 6B_4_), the medial projections only begin to become fragmented (red arrows in Figure 6B_4_). Taken together, the *ex vivo* brain culturing system that we have established here, can be used to study dynamic processes such as neuronal remodeling in up to a single cell resolution.

## Discussion

During pupal development, the brain undergoes massive neuronal remodeling events that include axon pruning as well as regrowth to form new adult specific connections. While these processes occur throughout the brain, including the mushroom body (Lee et al., 1999) and antennal lobe (Marin et al., 2005; Sweeney et al., 2011), these processes have never been imaged using time-lapse imaging hence our limited understanding of the dynamic cellular mechanisms. Here we present an *ex vivo* brain culture system in which pupal brains continue to develop in kinetics similar to normal development. Using this system we provide the first time-lapse sequences of axon pruning of MB neurons in up to a single cell resolution.

### Brain culturing as a method to image neurodevelopmental processes

Brain culturing has been previously used to study axon pathfinding (Gibbs and Truman, 1998) branching (Zschätzsch et al., 2014), remodeling (Brown et al., 2006; Prithviraj et al., 2012; Medioni et al., 2015) and regeneration following injury (Ayaz et al., 2008). So far, however, *ex vivo* culture methods that allow high-resolution analysis of the dynamics of developmental processes have described mostly short-term live imaging of the central brain (Siller et al., 2005). In some cases, studies have cultured and imaged brains for long periods of time but report that brain development is slowed by a factor of about two (Gibbs and Truman, 1998; Brown et al., 2006). While some setups are complex (Ayaz et al., 2008; Williamson and Hiesinger, 2010), others are simpler (Medioni et al., 2015). Here we carefully characterize different media components or culture procedures to develop a system that allows long-term brain culturing in which development proceeds in kinetics similar to *in vivo* using a relatively simple setup. For example, while in all previous time-lapse setups, biological glues such as laminin and polylysine were used to fix the brain into one location (Brown et al., 2006; Williamson and Hiesinger, 2010; Zschätzsch et al., 2014; Medioni et al., 2015), we found that this dramatically slowed down development, consistent with previous reports (Gibbs and Truman, 1998; Brown et al., 2006). We also tested mechanic immobilization of the brains in a ring apparatus that was made according to previously described larval brains culturing and imaging system (Siller et al., 2005), but while the setup was tricky we also found it does not support long-time culturing due to the low volume of culturing media contained in this device. Therefore, the main advantage of our system is that it provides a simple setup that allows long term time-lapse imaging in kinetics similar to what occurs *in vivo*.

Nonetheless, our culture system, as well as others, has some key limitations. First, dissection of brains at the onset of pupariation (0 h APF) led to little or no pruning in culture. This is consistent with previous studies (Prithviraj et al., 2012) and suggests that we still do not fully understand the signaling events that occur at the onset of pupariation. Yet, even culturing brains that were dissected from 5 h old pupae allows sufficient time to include the initial stages of γ axon pruning. Our study suggests that ecdysone concentrations need to be calibrated based on the developmental stage of interest. Second, in our hands imaging with an inverted confocal microscope was not efficient as it led to fast bleaching. Medioni et al. (2015), however, successfully used an inverted confocal with the ultra sensitive GaAsP detectors. This might provide a better solution when imaging of multiple fluorophores is needed. Finally, our setup allows only low throughput of live imaged brains. This is a result of the limited scanning speed of neurons that have elaborate branches over a thick brain slice in high resolution. Further adaptation of the mounting scheme combined with faster scanning, for example by using a spinning disk confocal with GaAsP detectors, might provide the means to overcome this limitation.

In sum, careful optimization of the culture conditions, and most importantly the varying requirements of ecdysone concentration, together with the immobilization of the brains without biological glues, has enabled us to develop a simple long-term *ex vivo* culture system that is suitable for time lapse imaging.

### The sequence of MB axon pruning includes blebbing and severing

Most cases of long-range neurite pruning, including MB remodeling, occur by localized fragmentation (Schuldiner and Yaron, 2015). Yet, the precise cellular mechanisms are mostly unknown. One exception is the dendrite pruning of the dendritic arborization (da) sensory neurons in *Drosophila* periphery where time-lapse imaging has shown that pruning is initiated by a severing event followed by fragmentation and engulfment by neighboring cells (Williams and Truman, 2005). While the fact that MB axons are eliminated by local degeneration was known (Watts et al., 2003) it was unknown whether it also involves a single severing event like da neurons. Due to some fundamental differences between dendrite pruning of da neurons and axon pruning of MB neurons, it was interesting to compare the cellular mechanisms underlying these processes (Yu and Schuldiner, 2014). We found that indeed axons bleb prior to the formation of one or more nicks that initiate the elimination of the distal part of the axon. Importantly, our data supports the formation of more than one axon severing event within the same cell. Interestingly, while imaging multi single-cell clones we observe that distinct axons eliminate at variable kinetics, as nicks were initiated at different time points and varied in their location. Furthermore, we have shown that the dorsal and medial extensions of the same neuron also degenerate at slightly different times.

### Future applications of live imaging to delineate various aspects of the cellular mechanisms of neuronal remodeling

The *ex vivo* brain culturing system that we have presented here should be useful to study many aspects of the cellular mechanisms of neuronal remodeling. For example, we would like to explore the dynamic relationship between neurons and glia during remodeling. While astrocytes were shown to be important for engulfment of axonal debris (Hakim et al., 2014; Tasdemir-Yilmaz and Freeman, 2014), whether glial cells in the CNS also provide spatial or temporal regulation during MB remodeling is not known. For these experiments we would need to expand our genetic toolbox by using multiple binary systems to visualize and manipulate MB neurons and glial cells at the same time.

Likewise, we are interested in investigating dynamic processes within remodeling MB neurons. For example, cytoskeletal dynamics could be studied by using fluorescence recovery after photobleaching (FRAP), or by utilization of photo-convertible proteins. In addition, it would be interesting to analyze calcium dynamics during γ axon remodeling by imaging various calcium sensors. In an elegant study, Kanamori and colleagues employed the genetically encoded calcium indicator GCaMP3 and showed that compartmentalized calcium transients provide temporal and spatial cues to induce dendrite pruning of *Drosophila* da neurons (Kanamori et al., 2013). However, whether the calcium transients are also important during MB axon pruning remains to be determined.

The brain culturing and time-lapse imaging method that we have presented should be useful to study dynamic neurodevelopmental processes occurring in other central brain neurons as well. For example, olfactory receptor (OR) axons and projection neuron (PN) dendrites meet and form synapses in the antennal lobe mostly during pupal development. However, the dynamics of this matching process are not known.

In addition to live-imaging, our *ex vivo* system also provides means to pharmacologically perturb different aspects of neurodevelopment (see for example, Rabinovich et al, submitted). Taken together, we anticipate that this *ex vivo* brain culturing and time-lapse imaging will complement the awesome power of fly genetics to better understand dynamic cellular events during development.

## Conflict of interest statement

The authors declare that the research was conducted in the absence of any commercial or financial relationships that could be construed as a potential conflict of interest.
