# Supplementary material for: Long term ex vivo culturing of Drosophila brain as a method to live image pupal brains: insights into the cellular mechanisms of neuronal remodeling
Source: Front Cell Neurosci. 2015 Aug 24;9:327. doi: 10.3389/fncel.2015.00327 (PMC4547045; doi:10.3389/fncel.2015.00327)
Supplement: Supplementary file 10 [file DataSheet1.PDF]

Supplementary Material

<u>Protocol for *ex vivo* drosophila brain time-lapse imaging</u>

<u>(This protocol describes our method using an upright microscope with a dipping lens – we used the Zeiss LSM 7 MP microscope with a Spectraphysics two-photon laser)</u>

- Important – Optimize culture conditions (Ecdysone concentration, for example) prior to time-lapse imaging experiments.
- Collect white pupae and age until the required developmental stage in a normal fly incubator at 25º.
- Disinfect all equipment using 70% ETOH (forceps, imaging chamber, molding tooth) and 100% ETOH (microscope objective-just before imaging).
- Prepare imaging chamber (half an hour before imaging).
    - Disinfect imaging chamber with 70% ETOH and air dry.
    - Melt 2% UltraPure LMP Agarose (Invitrogen 16520-050) in DDW at 65ºC.
    - Fill inner chamber (see Figure 4A) precisely up to its edge.
        - Important - over or under-filling the chamber might interfere with the molding step (see next).
    - Insert molding tooth (see Figure 4A).
        - This step is supposed to create a flat and embedded surface. If the chamber was not filled properly, then the tooth will not create a flat surface – under-filling will result in a non-flat surface while over filling will results in spill over that will interfere will the movement of the microscope lens.
- Prepare and filter culture media
        - We recommend keeping aliquots of the different ingredients at -20ºC and thawing only the required amount. In any case, do not refreeze.
- Dissect pupae in culture media
        - Take special care not to injure the brains and make sure to remove the imaginal discs completely – this is time consuming but in our experience significantly improves culturing outcome.
- Fill outer imaging chamber bath with the culture media.

- Carefully remove molding tooth
    - Important – prior to physically removing the molding tooth, delicately separate the agar from the tooth with forceps under a dissecting stereoscope.
    - If the agar disconnects from the chamber, it can be gently put back by using the handle of the forceps, to gently nudge the agar in place. Sometimes, making small perforations throughout the depth of the agar helps relieve trapped fluids at the bottom of the chamber.
- Transfer the desired brain onto the flat agar
- Use forceps to make indentations in the agar that will fit single brains.
    - Use the brains as size guides for digging the pits in the agar whilst making sure the pit is slightly smaller than the brain itself as to hold the brain fixed during the imaging.
    - The pits themselves should be at the center of the chamber for optimal lens positioning and movement.
- Guide the brain into the pit in the desired orientation and tap it in lightly to make sure it is secure in place.
    - It is possible to use the VNC as an anchor for the brain by sticking it in the agar itself, although this is quite aggressive and often damages the brain.
- Transfer the imaging chamber with the mounted brains to the microscope.
    - When transferring the brain from the dissecting stereoscope to the microscope, remove most of the fluid, as to prevent ripples caused by movement. Refill gently when the chamber is mounted on the microscope while making sure the brains do not float.
- Image immediately.
    - In any case try to keep the laser power to a minimum in order to avoid bleaching.
        - The more you zoom, the resolution will be better but you risk loosing the brain due to movement out of frame during imaging. Movement within the frame during the imaging can be fixed using Fiji plugins such as “stack-reg” and “correct 3D drift”.
    - For multi specimen imaging we used the Zen MTS macro. The amount of brains that can be imaged at once depends on the frequency of images you want to take (higher frequency, less brains).

- It is important to note that during the first few hours the brain settle into the agar and might move, mostly in the Z axis. Therefore we recommend that you track their position and correct if necessary.
